# Cretaceous gnetalean yields first preserved plant gum

**DOI:** 10.1038/s41598-020-60211-2

**Published:** 2020-02-25

**Authors:** Emily A. Roberts, Leyla J. Seyfullah, Robert F. Loveridge, Paul Garside, David M. Martill

**Affiliations:** 1 School of the Environment, Geography and Geosciences, University of Portsmouth, Burnaby Road, Portsmouth, PO1 3QL UK; 2 Department of Palaeontology, University of Vienna, Althanstraße 14, 1090 Vienna, Austria; 3 Conservation Research, The British Library, 96 Euston Road, London, NW1 2DB UK

**Keywords:** Biogeochemistry, Ecology

## Abstract

Some liquid plant exudates (e.g. resin) can be found preserved in the fossil record. However, due to their high solubility, gums have been assumed to dissolve before fossilisation. The visual appearance of gums (water-soluble polysaccharides) is so similar to other plant exudates, particularly resin, that chemical testing is essential to differentiate them. Remarkably, *Welwitschiophyllum* leaves from Early Cretaceous, Brazil provide the first chemical confirmation of a preserved gum. This is despite the leaves being exposed to water twice during formation and subsequent weathering of the Crato Formation. The *Welwitschiophyllum* plant shares the presence of gum ducts inside leaves with its presumed extant relative the gnetalean *Welwitschia*. This fossil gum presents a chemical signature remarkably similar to the gum in extant *Welwitschia* and is distinct from those of fossil resins. We show for the first time that a water-soluble plant exudate has been preserved in the fossil record, potentially allowing us to recognise further biomolecules thought to be lost during the fossilisation process.

## Introduction

A wide variety of vascular plants produce fluid exudates^1^ e.g. resins and gums, with each group differing in chemical definitions (Table 1). Due to similarity in physical appearance distinguishing exudates based on chemistry is vital, for example gums and resins are visually similar resulting in these terms being used interchangeably^1^. However, their chemical definitions are very different (Table 1); resins are composed of lipid-soluble terpenoids^1,2^, while gums are complex, highly branched (non-starch) water-soluble polysaccharides^3^. A common example of this misunderstanding is the *Eucalyptus* tree, which is known as a gum tree, but nuclear magnetic resonance analysis of the *Eucalyptus* exudate shows its composition to be polyphenolic and is therefore actually a kino^4^ (Table 1). Differences between gum and resin can also be seen in the functional roles within the plant. The main roles of resins are to respond to wounding, as a defence against pathogens and to dissuade herbivory by insects and other organisms^2,5,6^. Gum is involved in food storage, structural support, and also for wound sealing, but there is no common role across taxa^3^. Further confusion arises as some plants, e.g. *Boswellia* and *Commiphora* species, even produce exudates with a mixture of polysaccharide and resin components (the gum resins myrrh and frankincense respectively)^1^.

**Table 1:** Appearance, chemistry and preservation potential of plant exudates.

Exudate	Physical appearance	Chemical definition	Preservation potential
Resin	Yellow, amber or brown in colour. Hardens on exudation	Mostly composed of terpenoids and/or phenolic compounds	Some form ambers
Gum	White, yellow, amber or brown in colour. Hardens on exudation	Complex polysaccharides	First evidence presented here
Gum resin	Yellow, amber, or brown in colour. Hardens on exudation	Mixture of polysaccharides and terpenoids and/or phenolic	Unknown
Mucilage	Clear and gelatinous	Acidic or neutral polysaccharide polymers	Unknown
Kino	Red. Hardens on exudation	Polyphenolic	Unknown
Latex	White ‘’milky”, clear, orange or yellow in colour. Emulsion that coagulates on exudation	Terpenoids, proteins, carbohydrates, phenolics etc.	Preserved as fibres
Wax	White or clear in colour	Alcoholic esters of fatty acids	Preserved as part of the cuticle
Oil	Yellow or clear in colour	Fatty acids and glycerol	Unknown

Until now only fossilised plant resin (ambers)^7^ and latex filaments have been reported preserved in the fossil record^8,9^. While the fossilisation of fluid exudates might seem unlikely, the fossilisation of resin is relatively common, and extends back some 320 million years to the Carboniferous^10^, but chemically confirmed gums have never been reported.

The Early Cretaceous (~120 million year old) Crato Formation^11^ of northeast Brazil (Supplementary Fig. S1) is a well-known laminated limestone deposit that yields exceptionally preserved vertebrates, arthropods, and plants from the Nova Olinda Member^12^. Investigations of different groups of fossilized animals from the Crato Formation show that they are preserved as various mineral replacements, and their preservation was microbially-mediated^13–17^.

Amber has also previously been reported from the Crato Formation Lagerstätte, inside fossil plant remains and as isolated clasts^18–20^, and is attributed to conifers^18,19^. The fossil leaves occur as compressions showing at least some three-dimensionality (Supplementary Fig. S2). An amber-coloured substance is visible in some of the fossil leaves of *Welwitschiophyllum brasiliense* Dilcher *et al*. 2005 from the Crato Formation.

*Welwitschiophyllum* is considered a relative of the extant gymnosperm *Welwitschia mirabilis* Hooker 1863, the sole member of this gnetalean genus^21^. The Welwitschiaceae have a sparse macrofossil record, fossils assigned to this family, including *Welwitschiophyllum*, derive solely from the Crato Formation^21^. However, the pollen record shows Welwitschiaceae were once a diverse and prevalent group^22^ that saw a decline with increasing angiosperm pollen^23^. Today, *Welwitschia* is restricted to the Namib Desert in Namibia and Southern Angola and has chemically confirmed gum in both the cone and in abaxial ducts within leaves^24,25^.

We investigated this amber-coloured substance inside fossil *Welwitschiophyllum* leaves to test whether *Welwitschiophyllum* produced a resin (now fossilised as amber), or a gum like its presumed extant relative *Welwitschia*, using Fourier-transform infrared spectroscopy (FTIR) and Attenuated total reflectance (ATR) spectroscopy. We report here the first geochemical evidence for fossilised gum preserved inside *Welwitschiophyllum* leaves, and suggest areas for future investigation to understand how a ~120 million year old gum may have survived.

## Results

### *Welwitschiophyllum* leaves

**Figure 1:**
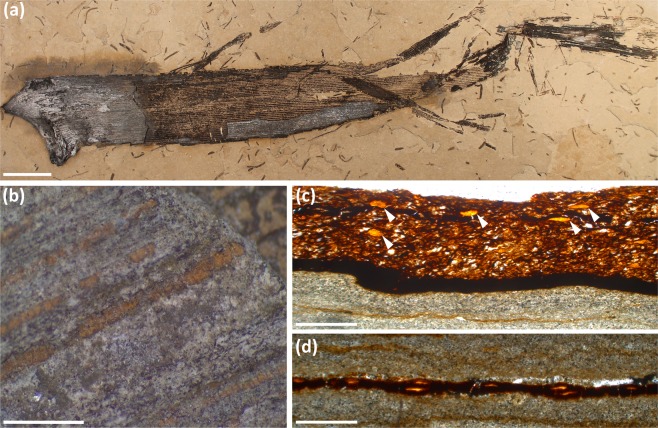
Fossil *Welwitschiophyllum* leaves with gum ducts from the Crato Formation, Brazil. (**a**) Complete elongate fossil leaf (UERJ 13-P1) showing a curved base and degraded fibrous apex, with a partially abraded surface (lighter part of fossil) exposing the internal leaf tissue and linear gum duct arrangement. (**b**) Detail from (**a**) (UERJ 13-P1) where the gum ducts appear as amber-brown structures within *Welwitschiophyllum* leaf tissue. (**c**) Transverse thin section through the fossil leaf (UERJ 14-P1) with arrowheads indicating the orange coloured gum ducts within the brown leaf tissue. The black line of tissue may be compressed remains of vascular tissue (below the leaf is the preserving sediment). (**d**) An oblique thin section of the leaf (UERJ 14-P1) showing the repeating pattern of the amber-coloured gum ducts. Scale bars, (**a**) 20 mm (**b**) 3 mm, (**c**) and (**d**) 500 μm.

Fossils of *Welwitschiophyllum* occur as long detached leaves up to 850 mm in length with thin bands of an amber-like substance. (Fig. 1a and Supplementary Fig. S2a). These are particularly visible where the fossil surface has been abraded or removed (Fig. 1b). This substance in the *Welwitschiophyllum* leaves resembles amber in ducts, lying parallel to the long axis of the leaves (Fig. 1b). These ducts are inferred here as adaxial (upper leaf surface) due to the curvature of the leaf base. However, the absence of preserved cuticle and other anatomical features, means that their precise orientation cannot be confirmed. This constituent arrangement contrasts with the traumatic formation of gum in its presumed relative *Welwitschia*. Slight compaction of the specimens gives these ducts an ellipsoidal cross section, (Fig. 1c and Supplementary Fig. S3) but they appear to have a repeating pattern showing a principal duct followed by a secondary duct ranging in diameter from 75 µm to 200 µm (Fig. 1d).

### Spectroscopy

**Figure 2:**
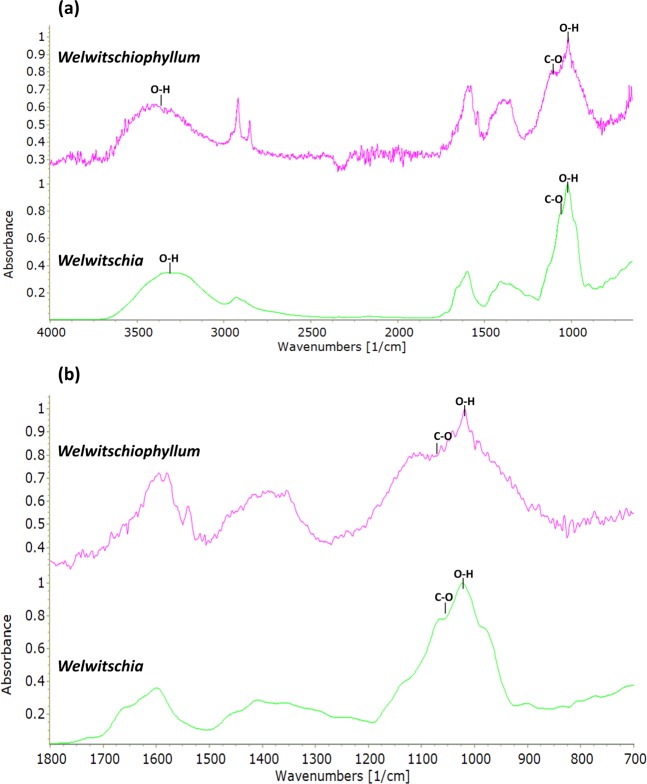
ATR spectra of the fossil gum extracted from *Welwitschiophyllum* (UERJ 13-P1) leaf, and gum from extant *Welwitschia*. (**a**) Overview of the ATR spectra with chemical bonds indicated. (**b**) Detail of the fingerprint regions of gums from both *Welwitschiophyllum* and *Welwitschia* with chemical bonds indicated.

Analysis using FTIR and ATR is commonly used on both living and fossil plants showing that complex biomolecules survive and are identifiable in the fossil record^26–28^. FTIR analyses compare living and fossil resin and gum samples (Supplementary Fig. S4). Additionally, ATR analysis confirms that the amber-coloured substance in the fossil leaves, which was extracted and purified for testing, generated a spectrum closely matching those of published gum signatures^26^ and is remarkably similar to that of *Welwitschia* gum (Fig. 2a). The ‘noise’ seen in the preserved gum spectrum (spectral line ‘waviness’ over the broader signal detected) was generated possibly due to the very small amount of material available for analysis. It is unlikely but also possibly due to the laser power and low accumulation numbers, but 32 accumulations were made per sample and no other sample showed this feature. Despite the noise, the key features of the spectrum are clearly visible. The diagnostic features of gums are a very large hydroxyl peak at ~3400 cm^−1^, peak absence at 1516 cm^−1^ and a very strong peak at 1077 cm^−1 26^. Using ATR these peaks can be seen both in the fossil *Welwitschiophyllum* and the extant *Welwitschia* with the peak at 1077 cm^−1^ appearing as a shoulder on a strong O-H stretch peak in *Welwitschiophyllum* (Fig. 2).

### Gum solubility

The discovery of *in situ* preserved plant gum is unusual because of its solubility in water. This is particularly striking in a formation thought to be deposited in a hypersaline lagoon setting. Solubility experiments were undertaken on *Welwitschia* gum to determine whether the increased salinity of the lagoon may have affected the solubility of the gum in any way (Supplementary Table S1). In the freshwater, brackish, normal marine, and hypersaline water tests the extracted gum dissolved within 49–59 minutes, showing that salinity does not affect solubility, and therefore the preservation (or not) of exposed gum.

## Discussion

Our analyses of the amber-coloured substance inside the fossil *Welwitschiophyllum* leaves shows a distinct chemical spectrum that clearly differs from those of ambers and resins^17,18^ (compare Supplementary Fig. S4 *Brachyphyllum* amber), but which closely compares to plant gum spectra, including our comparison sample (see Fig. 2) and those found in the literature^26^. This means that the recovered substance from the Crato Formation fossil *Welwitschiophyllum* leaves is a preserved gum and not an amber formed from resin. The chemically detected presence of gum in ducts inside two separate fossil leaves (Fig. 1 and Supplementary Fig. S3) confirms that this is not an isolated occurrence within these Crato Formation fossils.

Due to the soluble nature of gum, its preservation in the fossil record is unexpected. This is particularly notable here as the leaves containing gum were firstly deposited in a hypersaline lagoon, then later this deposit was exposed to continental weathering^18^. Thus, water featured in both the formation and weathering of the Crato Formation, yet the gum persisted. The gum solubility experiments showed that in each case of differing salinities the extracted *Welwitschia* gum dissolved, so saline levels appear to have no bearing on gum preservation.

How the gum came to be preserved is currently not understood and further investigation is needed into the taphonomic and diagenetic processes surrounding these gum-preserving fossil leaves. We can speculate that there are at least two factors involved. Firstly, the nature of the microbially-mediated taphonomy and diagenesis in the Crato palaeo-lake setting has been shown to be critical in the preservation of labile structures in animals from the Crato Formation Konservat-Lagerstätte^14–17^. Secondly, perhaps only in part, the coriaceous nature of the fossil leaves played a role. Both the surrounding duct tissue and the large amount of resistant embedding leaf tissues would have provided some protection from dissolution in water. In extant *Welwitschia* the outer walls of the epidermal cells are specialised with three layers, thickening and strengthening the epidermis^25^, but the preservation of the fossil leaves prohibits epidermal comparison. The regular arrangement of ducts in *Welwitschiophyllum* suggests that they were formed through duct initiation^29^, i.e. constituent, as opposed to the stress initiated response known as gummosis^30^. Their formation was likely to be used for food storage or structural support, signifying that the hydrophilic gum was constituent within the fossil leaves.

Despite the very low preservation potential of a highly water-soluble exudate, the first preserved gum was recovered from the Early Cretaceous. This fossil gum presents a chemical signature remarkably similar to gum in extant *Welwitschia* and distinct from those of fossil resins. This shows that gum production in plants extends back into the fossil record by at least ~120 million years. This is then the first report of a highly soluble biomolecule recovered from the Crato Formation and future work should focus on how this preserved gum survived. Furthermore, fossilised plants with observed internal ‘resins’ should be chemically confirmed in case further instances of gums or other types of plant exudate can be identified from the fossil record.

## Materials and Methods

### Image processing

Macro images were taken using Canon EOS 1100D. Images were made into combined figures using Inkscape.

### Petrographic thin sections

To examine the fossil plant histology petrographic thin sections were made using standard procedures and examined using a Leica DM750P microscope.

### Fossil material extraction and comparison samples

The fossil amber-coloured material for both FTIR and ATR analysis (from Specimen *Welwitschiophyllum* UERJ 13-P1 and *Brachyphyllum* UERJ15-P1) was mechanically extracted from the fossil leaf remains using sterile scalpel blades and dental picks under a Leica EZ4W stereomicroscope. The extracted samples were washed in absolute alcohol to minimise contamination. A sample of the limestone matrix from the fossil leaves was also mechanically extracted using sterile scalpel blades and prepared for comparison. All these fossil material samples were then ground into a fine powder using a pre-autoclaved and pre-sterilised glass micro-mortar and pestle (new one for each sample to avoid cross contamination). The resulting powder was then further checked microscopically for any visible impurities, and none were seen.

For comparative purposes analyses were performed on recent exudate from the extant gnetalean *Welwitschia mirabilis* Hooker, 1863 from the gardens of the South African National Biodiversity Institute, Pretoria (SANBI), and a commercial sample of sandarac was obtained. Reference samples (of sandarac) were washed in isopropanol. These samples were then ground into a fine powder using a pre-autoclaved and pre-sterilised glass micro-mortar and pestle (new one for each sample to avoid cross contamination). These separately prepared powdered modern and fossil exudates were then ready for the spectral analyses.

### Spectral analyses

Analysis of gums and resins was performed using FTIR spectroscopy on a *PerkinElmer ‘Spectrum 400’* spectrometer, fitted with an ATR sampling accessory (range 4000–550 cm^−1^, 32 accumulations, 4 cm^−1^ resolution). KBr pelletisation was not necessary for this machine setup and the pre-powdered samples were applied to the measurement area of the spectrometer with a new and autoclaved micro-spatula (one per measurement). The machine was cleaned by vacuuming off the measured samples thoroughly, then wiping with pure ethanol until all traces were removed and allowed to dry between measurements (test spectra were made to ensure no cross contamination occurred). Sample UERJ 13-P1 (amber-coloured fossil substance from *Welwitschiophyllum*) was averaged from four scans (with 32 accumulations per sample), the other samples (*Welwitschia*, *Brachyphyllum*, sandarac and limestone) had only 1 scan (with 32 accumulations per sample), and the peaks in all cases were normalised.

To confirm the results from the averaged multiple FTIR scans and provide more sensitive data, selected samples were further assessed by hot aqueous extraction and ATR. For ATR analyses (on *Welwitschia* and *Welwitschiophyllum* sample (UERJ 13-P1) a small amount of the sample was placed in 0.5 ml of water, which was heated to approximately 90 °C for 10 minutes. A drop of the residual liquid was placed on the ATR crystal, and the water allowed to evaporate, leaving a film of the extracted material on the crystal surface. A spectrum was recorded (with 32 accumulations, to improve the quality of the data, due to the very small amount of material under analysis) and peaks were normalised. Care was taken to thoroughly clean the ATR crystal between sample measurements using warm water and pure ethanol and allowed to dry. Test spectra were made to ensure no cross contamination was occurring between the measurement scans.

A baseline correction for both analyses with reference points at 3715 and 1800 cm^−1^ was performed. In all cases, the spectrographs were visualised using Spectra Gryph 1.2.10 software.

### Solubility experiments

These were completed using a Gallen Kamp magnetic stirrer regulator set at speed 4, *Welwitschia* gum was placed in freshwater (water-62.5 ml, gum weight-0.08 g), brackish (water- 62.5 ml,1.25 ppt, gum weight-0.08 g), normal marine (water-62.5 ml, 2.18 ppt, gum weight-0.08 g) and hypersaline (water-62.5 ml, 3.12 ppt, gum weight-0.08 g) water.

### CITES permit

Analysis of extant *Welwitschia mirabilis* gum was performed on samples obtained on CITES permit No. 152606.

## Supplementary information

Supplementary information.

Supplementary data.

Supplementary data.

Supplementary data.

Supplementary data.

Supplementary data.

Supplementary data.

Supplementary data.

Supplementary data.

Supplementary data.

## Data Availability

The fossil material from the Crato Formation examined here comprises three isolated leaves of *Welwitschiophyllum brasiliense* Dilcher *et al*. 2005 (Specimens UERJ 13-P1, UERJ 14-P1, and UOP-PAL-MC0002) and one specimen of *Brachyphyllum obesum* Heer, 1875. (UERJ 15-P1). Specimens UERJ 13-P1, UERJ 14-P1 and UERJ 15-P1 are accessioned at Rio Janeiro State University and UOP-PAL-MC0002 is accessioned at the University of Portsmouth. All data generated or analysed during this study are included in this published article (and its Supplementary Information files).
