# Supplementary material for: Cretaceous gnetalean yields first preserved plant gum
Source: Sci Rep. 2020 Feb 25;10:3401. doi: 10.1038/s41598-020-60211-2 (PMC7042272; doi:10.1038/s41598-020-60211-2)
Supplement: Supplementary file 1 — Supplementary information. [file 41598_2020_60211_MOESM1_ESM.pdf]

Cretaceous gnetalean yields first preserved plant gum

Emily A. Roberts[1,2*], Leyla J. Seyfullah[2], Robert F. Loveridge[1], Paul Garside[3] & David M. Martill[1]

[1]School of Earth and Environmental Sciences, University of Portsmouth, Burnaby Road, Portsmouth, PO1 3QL, UK

[2] Department of Palaeontology, University of Vienna, Althanstraße 14, 1090 Vienna, Austria

[3]Conservation Research, The British Library, 96 Euston Road, London NW1 2DB, UK.

Some liquid plant exudates (e.g. resin) can be found preserved in the fossil record. However, due to their high solubility, gums have been assumed to dissolve before fossilisation. The visual appearance of gums (water-soluble polysaccharides) is so similar to other plant exudates, particularly resin, that chemical testing is essential to differentiate them. Remarkably, *Welwitschiophyllum* leaves from Early Cretaceous, Brazil provide the first chemical confirmation of a preserved gum. This is despite the leaves being exposed to water twice during formation and subsequent weathering of the Crato Formation. The *Welwitschiophyllum* plant shares the presence of gum ducts inside leaves with its extant relative the gnetalean *Welwitschia*. This fossil gum presents a chemical signature remarkably similar to the gum in extant *Welwitschia* and is distinct from those of fossil resins. With exceptional preservation, we show that a water-soluble plant exudate can be preserved in the fossil record, potentially allowing us to recognise further biomolecules thought to be lost during the fossilisation process.

Supplementary Fig. S1. Stratigraphy of the Crato Formation. Stratigraphy of the Araripe Basin indicating the fossil bearing laminated limestones of the Nova Olinda Member of the Crato Formation. Stratigraphy modified from Martill et al.[1].

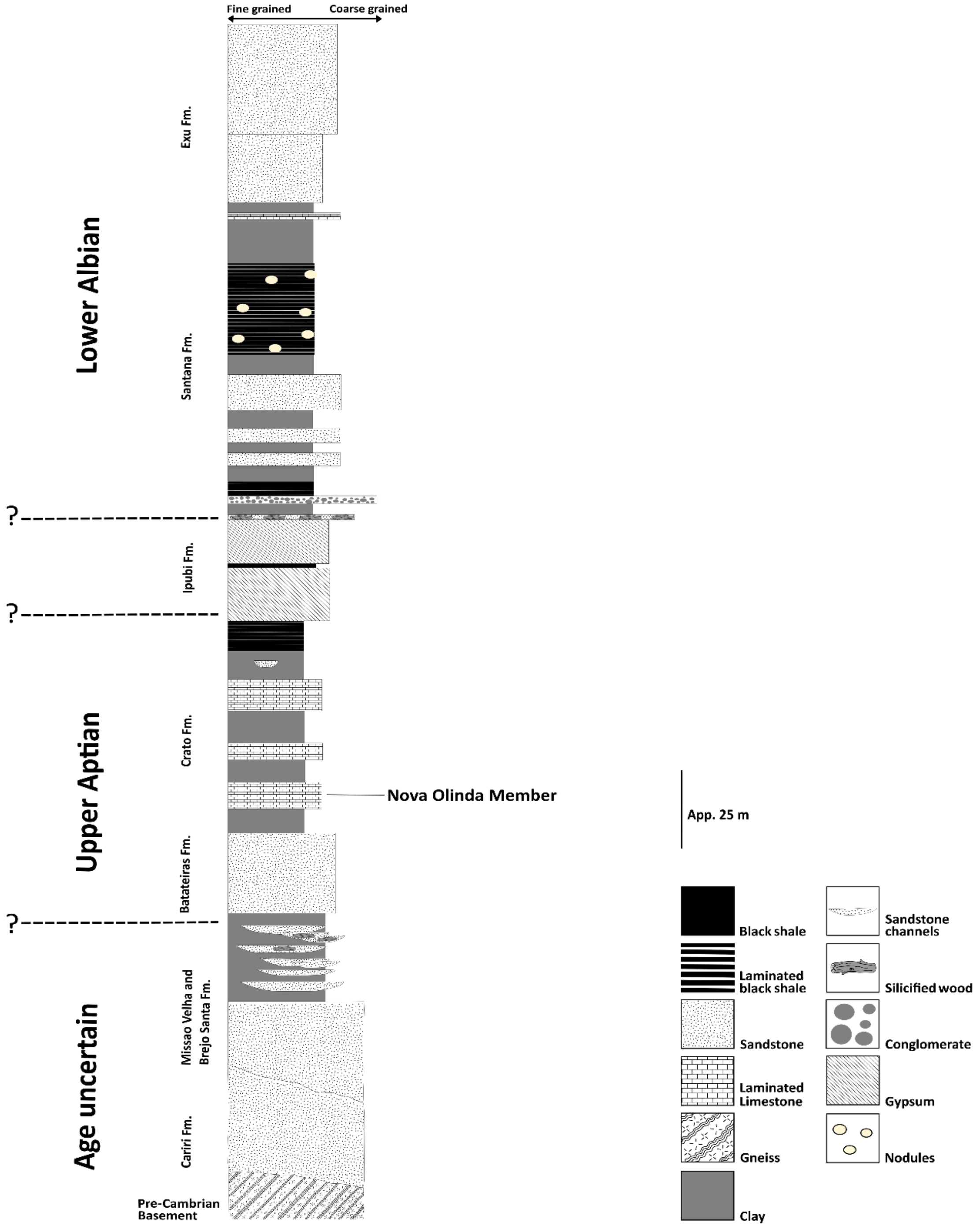

Supplementary Fig. S2. Fossil *Welwitschiophyllum* leaves from the Crato Formation, Brazil showing three- dimensional preservation. (**a**) Complete *Welwitschiophyllum* leaf (UOP-PAL-

MC0002). Showing three dimensionality. (**b**) A magnified view of *Welwitschiophyllum* (UERJ 14-P1) showing that the leaf is preserved three dimensionally with distinct parallel leaf tissues. Scale bars, (**a**) 10 mm (**b**) 5 mm.

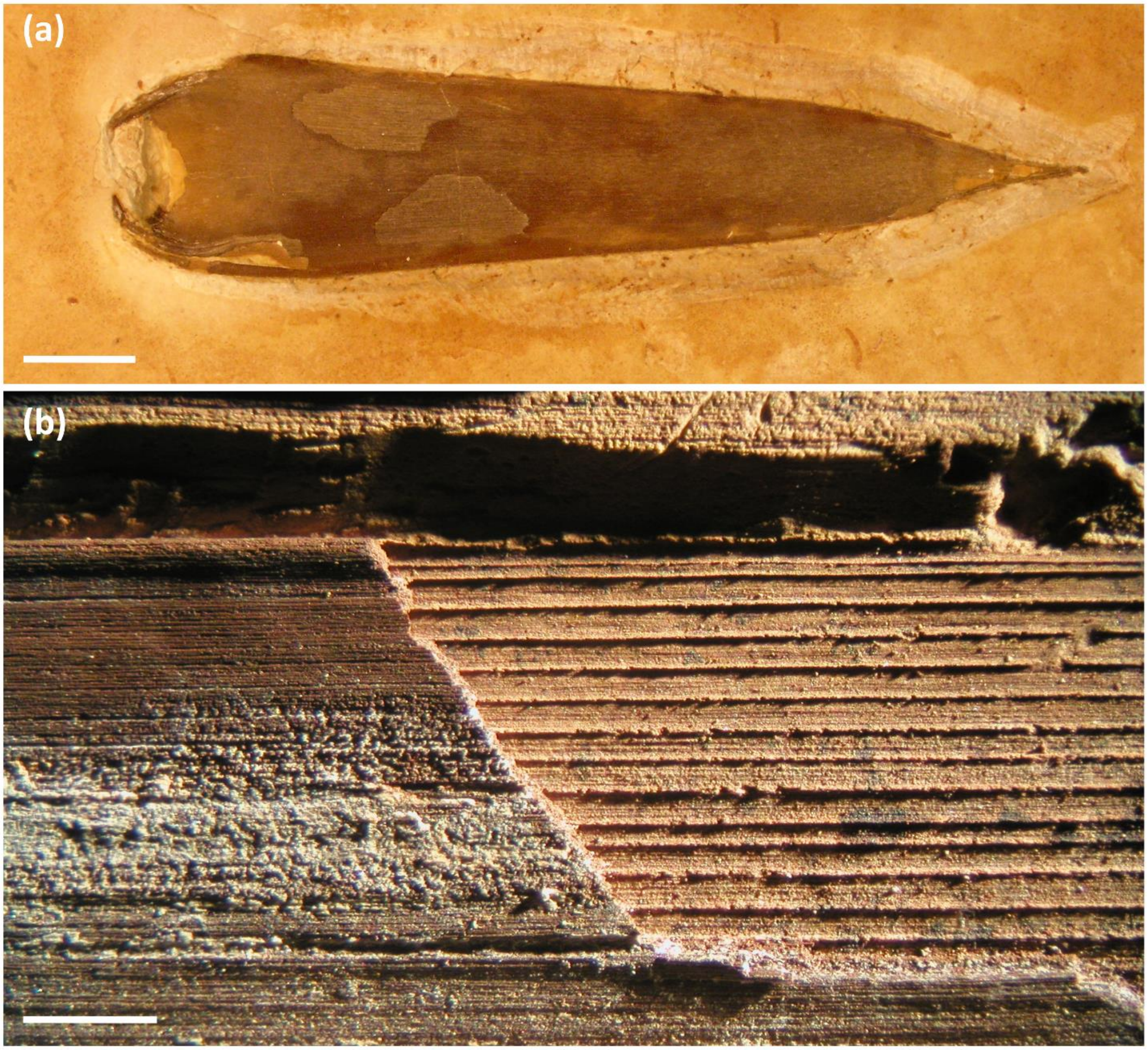

Supplementary Fig. S3. Thin section of a *Welwitschiophyllum* leaf. Thin section through the fossil leaf (UERJ 13-P1) showing amber-coloured gum ducts within brown leaf tissue. Scale bar, 500 μm.

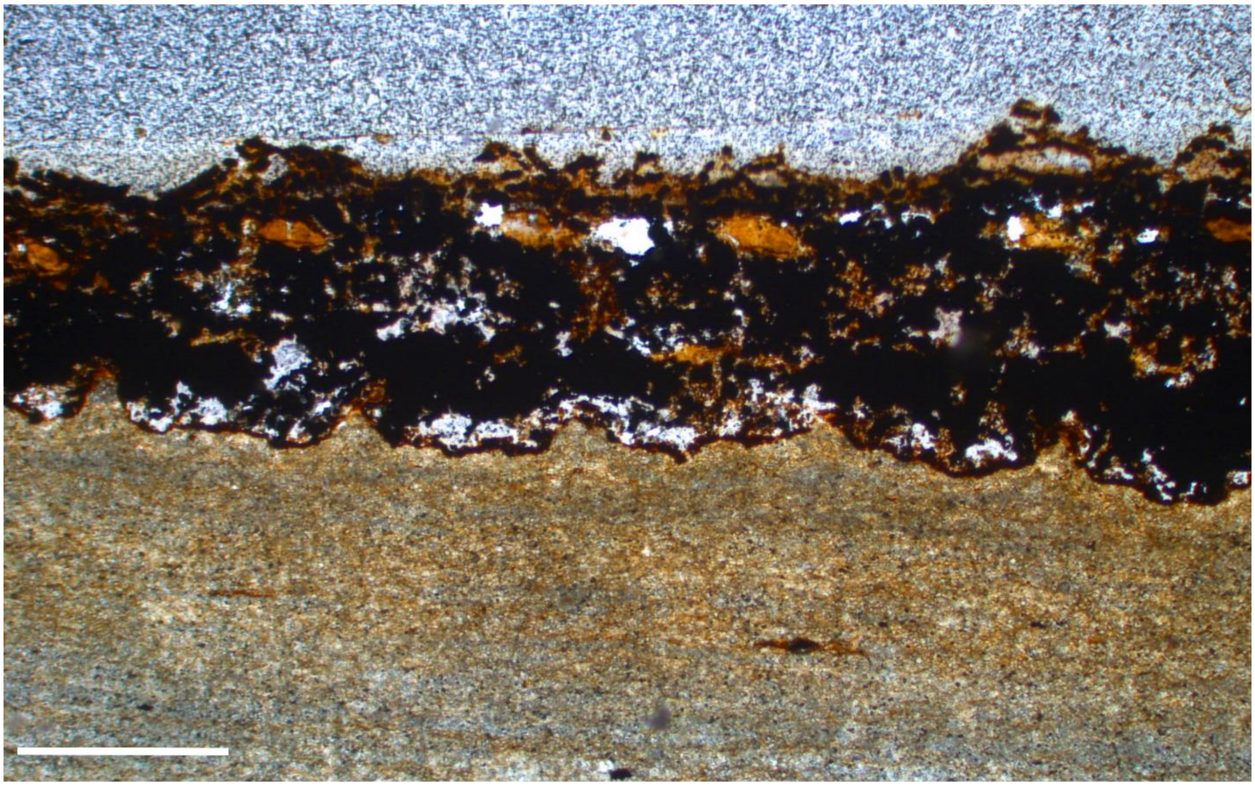

Supplementary Fig. S4. FTIR spectra comparing fossil *Welwitschiophyllum* gum to resin, limestone, and gum. (**a**) Crato limestone, *Welwitschiophyllum* gum (UERJ 13-P1), *Welwitschia*, sandarac (*Tetraclinis articulata)*, and *Brachyphyllum* amber samples (UERJ 15-Pl). The two peaks present at 2922 $cm^{-1}$and 2853 cm^{-1} in the *Welwitschiophyllum* spectra are not considered diagnostically important. The sandarac 'gum' and *Brachyphyllum* have spectra consistent with cupressaceous-type resin[26]; with diagnostic peaks at 1091 cm^{-1}, 1030 cm^{-1}, 886-889 cm^{-1} and 790-791 cm^{-1}). (**b**) Fingerprint region in detail showing Crato limestone matrix, *Welwitschiophyllum*, and *Welwitschia*. The dotted lines show sharp inorganic peaks at 873 cm^{-1} and 712 cm^{-1} present in both *Welwitschiophyllum* and the limestone matrix that surrounds the fossil, indicating only slight contamination of the fossil gum sample by the matrix. *Welwitschiophyllum* and *Welwitschia* share a diagnostic peak (1077 cm^{-1})

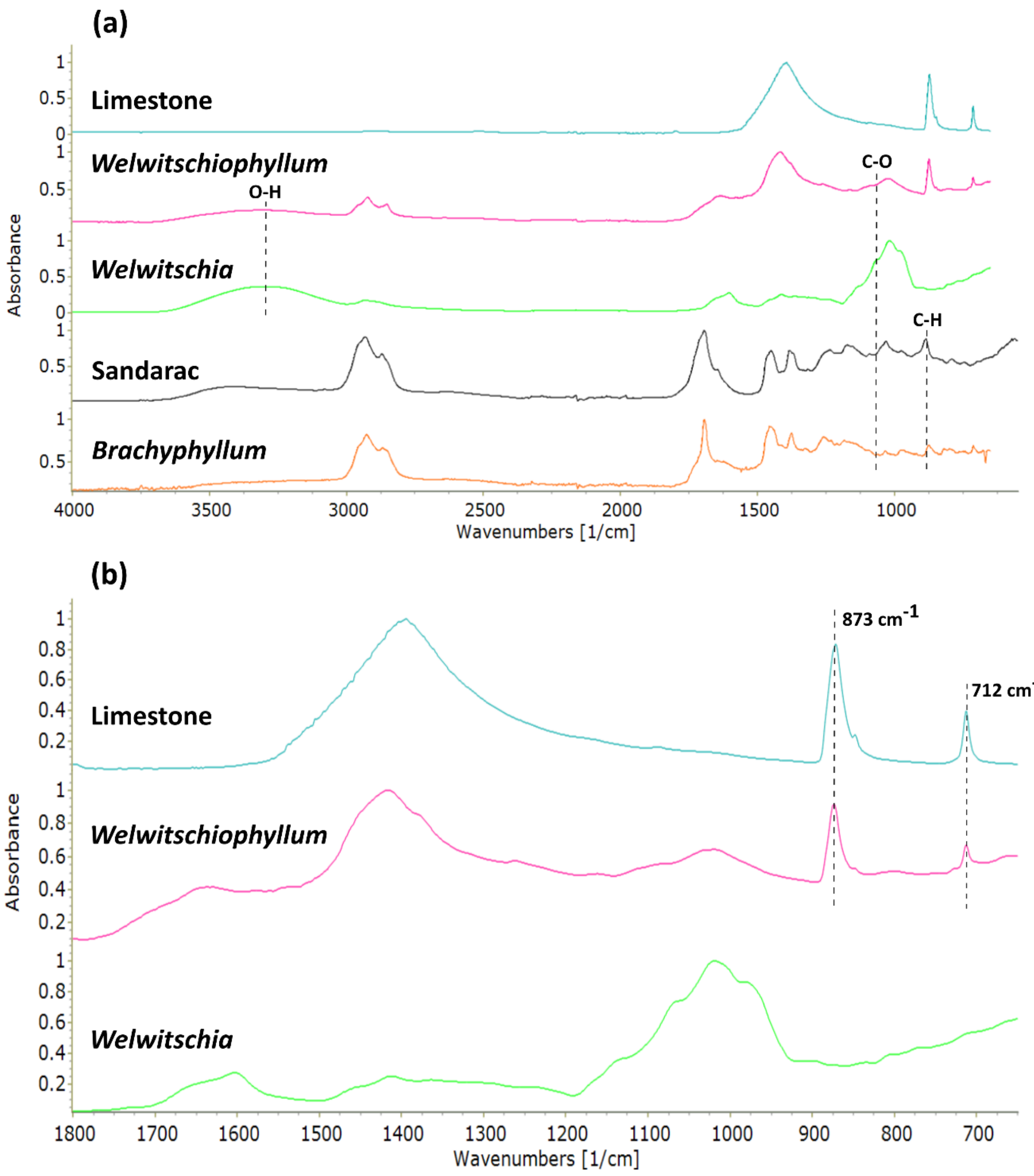

Supplementary Table S1. Solubility experiments

Salinity	Time taken to dissolve	Gum weight	Water
Fresh	51 minutes	0.08 g	62.5 ml
Brackish (1.25 ppt)	59 minutes	0.08 g	62.5 ml
Normal marine (2. 18 ppt)	49 minutes	0.08	62.5 ml
Hypersaline (3.12 ppt)	50 minutes	0.08g	62.5 ml

References

1. Martill, D. M., Loveridge, R. F., Mohr, B. A. R. & Simmonds, E. A. Wildfire origin for terrestrial organic debris in the Cretaceous Santana Formation Fossil Lagerstätte (Araripe Basin) of north-east Brazil. *Cretaceous Research* **34**, 135–141 (2012).
